# Tumour-Infiltrating Lymphocytes, Tumour Cell Density, and Response to Neoadjuvant Short-Course Radiotherapy in Rectal Cancer: A Translational Sub-Study from the MRC CR07 Clinical Trial

**DOI:** 10.3390/cancers17183040

**Published:** 2025-09-17

**Authors:** Jonathan P. Callaghan, Ross Jarrett, Alice C. Westwood, Jon Laye, Philip Quirke, Derek R. Magee, Daniel Bottomley, David Sebag-Montefiore, Lindsay Thompson, Angela Meade, Heike I. Grabsch, Nicholas P. West

**Affiliations:** 1 Division of Pathology and Data Analytics, Leeds Institute of Medical Research, University of Leeds, Leeds LS9 7TF, UK; j.p.callaghan@leeds.ac.uk (J.P.C.);; 2 School of Computing, University of Leeds, Leeds LS2 9JT, UK; d.r.magee@leeds.ac.uk; 3 Division of Oncology, Leeds Institute of Medical Research, University of Leeds, Leeds LS9 7TF, UK; 4 MRC Clinical Trials Unit, University College London, London WC1V 6LJ, UK; 5 Department of Pathology, GROW—Research Institute for Oncology and Reproduction, Maastricht University Medical Centre+, 6229 HX Maastricht, The Netherlands

**Keywords:** tumour infiltrating lymphocyte density, tumour cell density, neoadjuvant radiotherapy, rectal cancer

## Abstract

**Background**: Rectal cancer is common and frequently treated with neoadjuvant radiotherapy prior to surgery to reduce the risk of tumour recurrence. However, the therapeutic benefits and side effects of radiotherapy can vary between patients, and there are currently no validated biomarkers to predict treatment response. Tumour cell density (TCD) and tumour-infiltrating lymphocyte (TIL) density are proven prognostic biomarkers in colorectal cancer; however, their utility in predicting radiotherapy response remains unclear. We assessed the prognostic and predictive value of TCD and TIL density in rectal cancer patients treated with radiotherapy. **Methods**: TCD was quantified using a manual point-counting method in 253 pre-treatment biopsies and across the entire tumour area of 569 resection specimens from the MRC CR07 clinical trial, which randomised patients to either neoadjuvant short-course radiotherapy (SCRT) or straight to surgery (control). TIL density was measured in 102 biopsies and matched resection specimens (73 SCRT, 29 control) across different tumour areas using deep learning-based cell detection in MIM (HeteroGenius Ltd., Leeds, UK). Cutoffs for low/high-TCD and TIL density were both pre-defined and derived from survival data using the survminer R package. Survival analyses were performed to evaluate the predictive and prognostic value of TCD/TIL in relation to overall and cancer-specific survival. **Results**: TCD in the resection specimens was lower in the SCRT group (19.9%, IQR 12.9–26.7%) than the control group (34.3%, IQR 27.7–40.5%, *p* < 0.001). In control resections, low-TCD was associated with a higher risk of all-cause mortality (HR 2.20, 95% CI 1.41–3.44, *p* < 0.001) and cancer-related death (HR 2.69, 95% CI 1.41–5.13, *p* = 0.0026). In contrast, after SCRT, low resection TCD was associated with a reduced risk of death (HR 0.63, 95% CI 0.40–0.98, *p* = 0.04). In the SCRT group, low biopsy TCD prior to radiotherapy was associated with a reduced risk of cancer-related death (HR 0.34, 95% CI 0.13–0.89, *p* = 0.028). Across both trial arms, TIL density was higher in pre-treatment biopsies than resections (2492 vs. 1304/mm^2^, *p* < 0.001). Low biopsy TIL density was associated with an increased risk of all-cause mortality (HR 2.43, 95% CI 1.24–4.76, *p* = 0.01). The SCRT group had lower TIL density in the resection compared with controls (1210 vs. 1615/mm^2^, *p* < 0.001), and low resection TIL density across the whole tumour area was associated with a higher risk of death (HR 2.55, 95% CI 1.11–5.87, *p* = 0.027). **Conclusions**: Our findings support the role of TCD and TIL density as quantitative biomarkers in rectal cancer patients. TCD can be used to assess the degree of response to radiotherapy, and contrasting survival associations are observed between straight-to-surgery and SCRT-treated patients. This study raises the possibility of using TCD as both a prognostic and predictive biomarker. TIL density failed to show predictive value but demonstrated expected prognostic associations.

## Simple Summary

Measuring the density of tumour cells and immune cells in rectal cancer specimens can provide valuable insights into prognosis and help inform clinical management. Preoperative radiotherapy is commonly used in patients with rectal cancer to reduce the risk of recurrence. However, there is currently no reliable method to predict how an individual patient’s tumour will respond to radiation. This study aimed to investigate whether tumour cell or immune cell density in diagnostic rectal cancer biopsies could serve as a predictive marker for radiotherapy response. Using manual counting of tumour cells and an artificial intelligence tool to analyse routine pathology slides for lymphocytes in the tumour microenvironment, we found that radiotherapy reduced both the number of tumour cells and lymphocytes after treatment. Patient survival varied according to the density of tumour cells and lymphocytes in the pre-treatment biopsy. The findings suggest that quantifying tumour and immune cell density at diagnosis could support a more personalised treatment strategy for rectal cancer patients and potentially improve outcomes.

## 1. Introduction

Colorectal cancer represents a major global health burden, ranking as the third most commonly diagnosed cancer and the second leading cause of cancer-related death worldwide [1]. Rectal cancer accounts for a significant proportion of these cases and is typically managed using a multidisciplinary approach that combines surgery, radiotherapy, and chemotherapy depending on the disease stage and patient characteristics. Preoperative radiotherapy has been shown to reduce local recurrence and improve survival in resectable rectal cancer [2]. However, oncological benefits of radiotherapy are not universal [3], and treatment can lead to significant morbidity, including faecal incontinence and sexual dysfunction [4]. This variability in response underscores the need for robust and reliable biomarkers to identify patients most likely to benefit from neoadjuvant radiotherapy.

The Medical Research Council (MRC) CR07 trial was an international, multi-centre randomised controlled trial comparing short-course preoperative radiotherapy (SCRT) with selective postoperative chemoradiotherapy [5]. The availability of comprehensive follow-up data, paired pre- and post-treatment specimens, and a control group offers an ideal platform to investigate potential prognostic and predictive biomarkers of response and survival. Tumour cell density (TCD) and tumour-infiltrating lymphocyte (TIL) density have emerged as potential prognostic biomarkers in colorectal cancer patients not treated with neoadjuvant therapy [6,7]; however, their relevance in radiotherapy-treated rectal cancer remains unclear.

Tumour cells exist within a complex microenvironment where a lower tumour–stroma ratio has been linked with poorer overall and disease-free survival in several solid cancer types [8], including colon [9] and rectal cancer [10]. A previous study has linked a low-TCD (i.e. stroma-rich tumours) in colorectal cancer to poorer cancer-specific survival [6].

TILs are similarly well-established as prognostic biomarkers across multiple cancer types [11], including rectal cancer [12]. High densities of CD8-positive T lymphocytes within the tumour centre and invasive margin have been shown to predict lower rates of recurrence and improved survival in colorectal cancer [13]. In colorectal cancer, a high Immunoscore (based on CD3 and CD8 expression) is associated with better survival [14]. Neoadjuvant radiotherapy has also been shown to increase the number of CD8-positive cells in rectal cancer, reflecting robust immune activation [12]. Whilst immunohistochemistry for specific T cell subsets is informative [12,15], it can be expensive and time-consuming and may deplete valuable tissue. By contrast, quantifying TCD and TILs on routine haematoxylin and eosin (H&E)-stained tissue sections using digital pathology offers a quicker, more cost-effective, and scalable alternative for rapid implementation into clinical workflows. Automated, deep learning-based quantification of TILs on H&E slides has been applied in other cancer types, such as melanoma [16] and breast cancer [17], and a recent study suggested TIL density might predict adjuvant chemotherapy response in gastric cancer [18].

Given the current limitations in predicting radiotherapy outcomes, this study aimed to quantitatively assess TCD and TIL density in pre-treatment biopsies and post-treatment resection specimens from patients enrolled in the MRC CR07 trial and explore associations with survival, with consideration given to other known prognostic clinicopathological variables. Where possible, pre-defined cutoff values for dichotomisation were evaluated. We hypothesised that pre-treatment measurements of TCD and TIL density would hold prognostic value in rectal cancer and might predict response to neoadjuvant radiotherapy. If validated, these biomarkers could enable more personalised treatment selection by identifying patients likely to benefit most from preoperative radiotherapy and sparing others from unnecessary morbidity by selecting alternative therapeutic approaches.

## 2. Materials and Methods

### 2.1. Patient Cohort

Pre- and post-treatment pathological specimens from patients recruited to the MRC CR07 trial were analysed in this study. CR07, which recruited rectal cancer patients between 1998 and 2005, was a multi-centre, international, randomised controlled trial comparing short-course radiotherapy (SCRT) to selective postoperative chemoradiotherapy in patients with resectable rectal cancer [5]. Inclusion criteria specified that all patients had histologically confirmed adenocarcinoma of the rectum with no evidence of distant metastasis. In total, 1350 patients were recruited across 80 centres in four countries (UK, Canada, South Africa, and New Zealand). The SCRT regimen comprised 25 Gy in five consecutive daily fractions followed by surgery, recommended to take place within 7 days of the last fraction of radiotherapy. Pathological specimens were retrospectively requested from trial recruitment sites and included both formalin-fixed paraffin-embedded (FFPE) tissue blocks and glass slides from the diagnostic biopsy and surgical resection specimens, where available. Clinical data and patient follow-up were provided by the MRC Clinical Trials Unit.

### 2.2. Sample Preparation

Routine haematoxylin and eosin-stained tissue sections were received from participating centres or, where not available, were freshly prepared at four-micrometre thickness from FFPE blocks using standard laboratory protocols. All slides were scanned at ×20 magnification (Leica Aperio AT2 scanner, Aperio Technologies, Vista, CA, USA). Images were uploaded to and viewed in Medical Image Manager (MIM), a web-based platform for digital pathology image analysis (HeteroGenius Ltd., Leeds, UK). A single representative slide with tumour was selected from the diagnostic biopsies and surgical resection specimens for analyses.

### 2.3. Tumour Cell Density Assessment

Tumour cell density (TCD) was calculated across the entire tumour area using a method similar to one previously described [6]. In brief, the entire tumour area was annotated to include tumour-associated stroma and fibrosis. Approximately 300 points (±15) were systematically randomly allocated to the annotated area, and the tissue component at each point was manually determined by a trained observer. TCD was expressed as the percentage of points falling on viable tumour cells. In addition to the measurement of the entire tumour area in resections, TCD was also measured in biopsies in annotated areas of viable tumour. As an exploratory endpoint, the histological response to radiotherapy was also estimated through the absolute change in TCD (biopsy TCD minus whole TCD). A pre-defined TCD cutoff point of 47% [6] was initially used to stratify patients into high- and low-TCD groups in addition to exploring survival analysis-derived cutoff points.

### 2.4. Tumour-Infiltrating Lymphocyte Density Assessment

TIL density was calculated for all patients in the SCRT arm with available matched biopsy and resection slides. In addition, 32 patients from the control arm with matched biopsy and resection slides were analysed to investigate whether TIL densities were comparable in the straight-to-surgery population.

Digital slides were manually annotated in MIM according to a standardised protocol, similar to one previously described [19], and each image was independently reviewed by a second observer. Annotation regions in the resection specimen slide included the entire tumour area, tumour at the luminal surface, tumour core, and tumour at the invasive margin. Regions with normal tissue, low-grade dysplasia, significant haemorrhage, or necrosis were excluded from the annotation. As with the TCD assessment, the entire tumour area annotation included any tumour-associated stroma and radiotherapy-induced fibrosis or inflammation. The whole tumour area was used as a guide for the other annotation regions: up to five equidistant 2 mm diameter circles were placed along the luminal surface (luminal surface); up to two 3 mm diameter circles were placed in the area of greatest tumour cellularity (tumour core) and up to five 1 mm diameter circles were placed straddling the interface between tumour and normal tissue at the deep margin (invasive margin).

**Figure 1:**
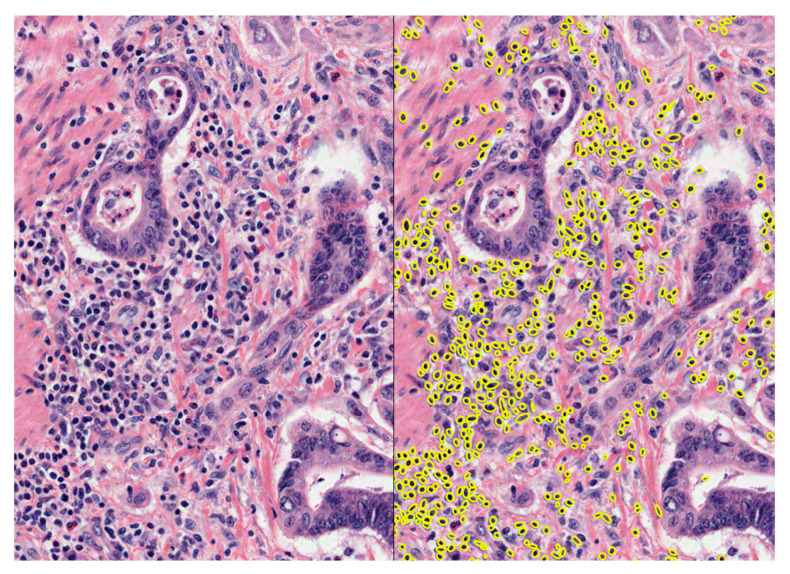
Haematoxylin and eosin-stained tissue section of colorectal cancer (**left**) and the same region with tumour-infiltrating lymphocytes (yellow annotations) identified through the cell analysis model in MIM (**right**).

TIL density was determined by deep learning using the cell analysis unit in MIM (Figure 1), which utilises a U-shaped encoder–decoder network architecture (UNET)-based cell detector to detect, segment, and classify cells by type in whole-slide images [20]. The model used for this analysis was trained on approximately 55,000 annotated cells from a mixture of oesophagogastric and colorectal cancer cases for 67,000 epochs [19]. No distinction was made between TILs within the stromal or epithelial compartments. The model was not trained to detect specific TIL subsets. TIL density was calculated per mm^2^ by dividing the total number of identified lymphocytes within an annotated region by the area of the annotation region. Where multiple annotation regions were made on the same image, the TIL densities in these regions were averaged across the number of regions.

### 2.5. Statistical Analysis

Given the non-normal distribution of TCD and TIL density, descriptive statistics are presented as median (interquartile range) and non-parametric tests were utilised for group comparisons. The relationships between TCD, TIL density, and various categorical clinicopathological variables (age group, sex, TNM stage, and trial arm) were assessed using Wilcoxon rank-sum and Kruskal–Wallis tests as appropriate. Differences between the biopsy and resection specimens were restricted to the patients with matched samples.

Overall survival (OS) and cancer-specific survival (CSS) were evaluated using the Kaplan–Meier method and log-rank tests. These evaluated survival probabilities over time for groups defined by TCD or TIL density categories of high vs. low, based on either pre-defined or survival data-derived cut-points. These optimal cut-points were generated using the surv_cutpoint() function from the R package survminer (version 0.5.1) [21], which identifies the cut-point yielding the most significant difference in survival curves between the groups. Cox’s proportional hazards regression was used to estimate hazard ratios (HRs), associated 95% confidence intervals, and *p*-values. Key clinicopathological variables, TCD, and TIL density were evaluated in univariate and multivariate models to determine their independent prognostic value with respect to OS. Patients with missing data for any of the variables were excluded from multivariate analyses. The median follow-up time for survival was 4068 days. Analyses focused on OS and CSS owing to the low numbers of local disease recurrence events in the trial.

TIL density comparisons were conducted between and within the two trial arms. Wilcoxon rank-sum tests assessed baseline differences in biopsy TIL density between control and treatment arms and evaluated treatment effects within specific tumour regions. Paired Wilcoxon tests compared biopsy TIL density with annotated tumour regions in resections within each arm. *p*-values of less than 0.05 were considered statistically significant.

Statistical analyses were conducted in R (4.4.2) using packages readxl (1.4.3), dplyr (1.1.4), survminer (0.5.0), survival (3.7-0), and ggplot2 (3.5.1).

## 3. Results

### 3.1. Patient Clinicopathological Characteristics

Out of 1350 patients recruited to the CR07 trial, in total, resection slides and/or biopsy slides were available for 604 (45%) patients from 41 centres. TCD was measured in the resection specimens of 569 patients (SCRT *n* = 275, control *n* = 294). In total, 12 resections were excluded due to the absence of a tumour or a lack of embedding the full thickness of the tumour. TCD was measured in biopsy specimens of 253 patients (SCRT arm *n* = 128, control arm *n* = 125). In total, 102 patients (SCRT arm *n* = 73, control arm *n* = 29) had matched biopsy and resection slides for the TIL density analysis. The clinicopathological characteristics of the patients are provided in Table 1. There was no relationship between resection TCD or resection TIL density measured in the whole tumour area and age group, sex, and (y)pT or (y)pN category. However, rectal cancers from patients in the SCRT arm showed significantly lower TCD and TIL densities when compared to the control arm (Table 1).

### 3.2. Tumour Cell Density

#### 3.2.1. Biopsy Tumour Cell Density

**Figure 2:**
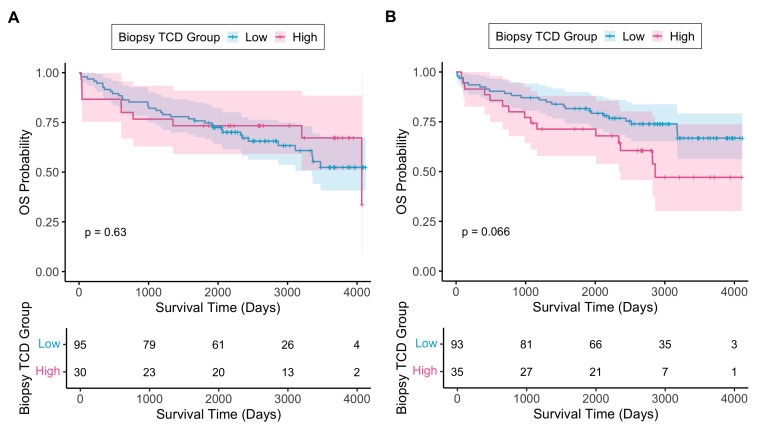

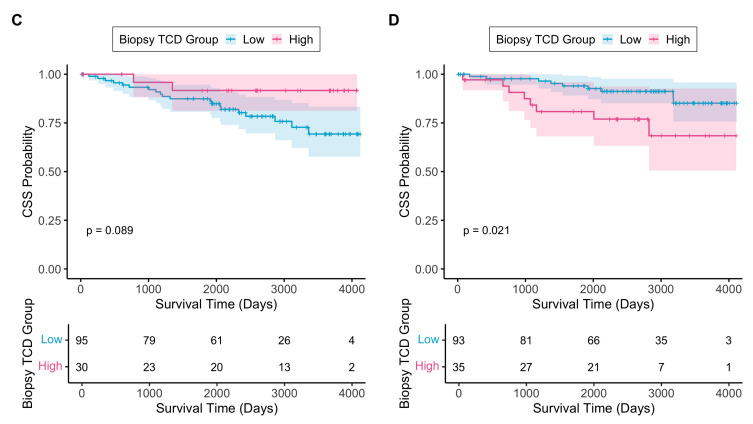
(**A**) Kaplan–Meier curve showing overall survival with respect to biopsy TCD low/high groups in control arm; (**B**) Kaplan–Meier curve showing overall survival with respect to biopsy TCD low/high groups in SCRT arm; (**C**) Kaplan–Meier curve showing cancer-specific survival with respect to biopsy TCD low/high groups in control arm; (**D**) Kaplan–Meier curve showing cancer-specific survival with respect to biopsy TCD low/high groups in SCRT arm. TCD cutoff points were 47.5% and 44.2% for control and SCRT arms, respectively. The shaded regions around each line represent 95% confidence intervals. The *p*-values on each graph are derived from the respective log-rank test. Beneath each graph is the respective number at risk table. Time zero represents the date of surgery.

The median biopsy TCD in the SCRT arm (36.4%, IQR 27.3–44.5%) was comparable to that of the control arm (36.9%, IQR 23.9–46.2%, *p* = 0.749). Figure 2 shows OS and CSS analyses for the control and SCRT arms using optimised TCD cutoff points. These novel cutoff points (47.5% and 44.2% in the control and SCRT arms, respectively) were derived from survival data to produce the greatest separation between low-TCD and high-TCD groups. In the control arm, no association was seen between low biopsy TCD and OS (HR 1.19, 95% CI 0.59–2.40, *p* = 0.631); however, low biopsy TCD did show a non-significant trend towards poorer CSS (HR 3.29, 95% CI 0.77–14.09, *p* = 0.109). In the SCRT arm, low biopsy TCD was associated with improved CSS (HR 0.34, 95% CI 0.13–0.89, *p* = 0.028) and a trend towards improved OS (HR 0.55, 95% CI 0.29–1.05, *p* = 0.070).

#### 3.2.2. Resection Tumour Cell Density Across the Whole Tumour Area

As expected, median whole tumour TCD in the SCRT arm (19.9%, IQR 12.9–26.7) was significantly lower than that seen in the control arm (34.3%, IQR 27.7–40.5, *p* < 0.001), Table 1.

**Figure 3:**
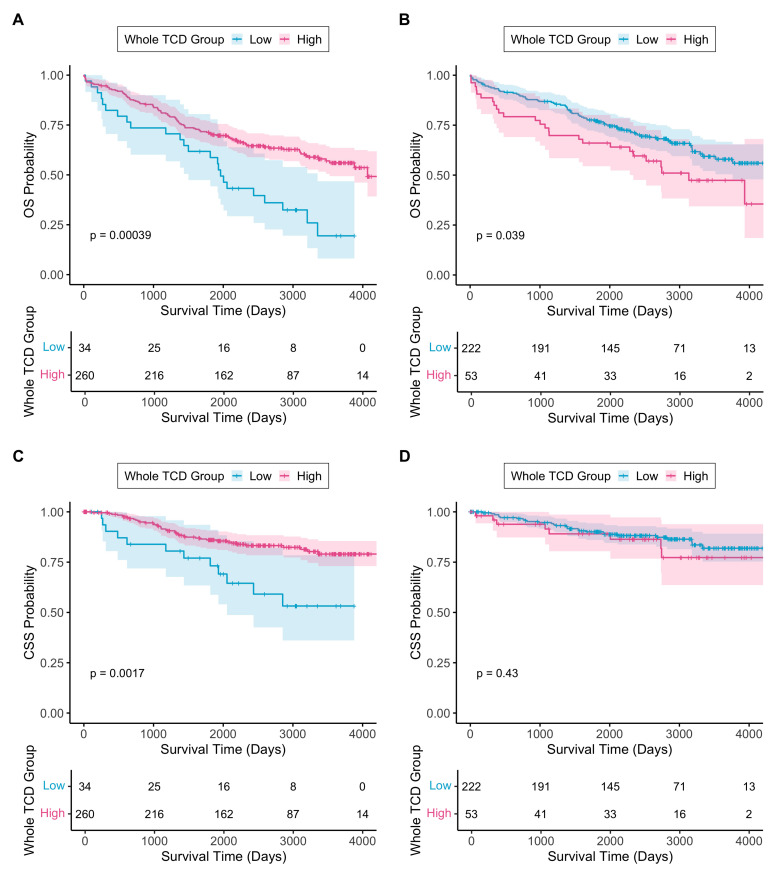
(**A**) Kaplan–Meier curve showing overall survival with respect to whole TCD low/high groups in control arm; (**B**) Kaplan–Meier curve showing overall survival with respect to whole TCD low/high groups in SCRT arm; (**C**) Kaplan–Meier curve showing cancer-specific survival with respect to whole TCD low/high groups in control arm; (**D**) Kaplan–Meier curve showing cancer-specific survival with respect to whole TCD low/high groups in SCRT arm. TCD cutoff points were 21% and 28.5% for control and SCRT arms, respectively. The shaded regions around each line represent 95% confidence intervals. The *p*-values on each graph are derived from the respective log-rank test. Beneath each graph is the respective number at risk table. Time zero represents the date of surgery.

Using a previously defined prognostic cutoff point of 47% from luminal TCD measurements of a straight-to-surgery population with colorectal cancer [6] showed that all patients from the current study would be classified as the low-TCD group. The survival data of the current cohort was used to calculate a novel TCD cutoff point separately for each trial arm (control arm: 21%, SCRT arm: 28.5%). In the control arm, low-TCD was associated with worse OS (HR 2.20, 95% CI 1.41–3.44, *p* < 0.001) and CSS (HR 2.69, 95% CI 1.41–5.13, *p* = 0.0026), as seen in Figure 3. In the SCRT arm, low-TCD after radiotherapy was associated with better OS (HR 0.63, 95% CI 0.40–0.98, *p* = 0.040), and no association was seen with CSS (HR 0.73, 95% CI 0.33–1.61, *p* = 0.436), as seen in Figure 3.

#### 3.2.3. Change in TCD Between Biopsy and Resection Specimens

There was no significant difference in TCD when analysing paired diagnostic biopsy and surgical resections in the control group (n = 120, median biopsy TCD was 36.9% (IQR 23.9–46.2%); median whole tumour area TCD was 34.9% (IQR 28.3–40.2%), *p*= 0.549). However, at the individual level, only 21 of 120 (17.5%) control arm patients had biopsy and resection TCDs that were within 5% of one another. There was a significant difference in TCD when analysing paired diagnostic biopsy and surgical resections in the SCRT group (n = 123, median biopsy TCD was 36.4% (IQR 27.3–44.5); median whole tumour area TCD was 20.3% (IQR 12.0–26.8%), *p* < 0.001).

Median absolute change in TCD (biopsy TCD minus whole tumour area TCD) was 0.39% (IQR−9.77 to 12.9%) in the control arm and 15.3% (IQR 6.23 to 27.4%) in the SCRT arm (*p* < 0.001), confirming that SCRT significantly reduced the TCD.

#### 3.2.4. TCD as an Independent Prognostic Biomarker

In univariate analyses, a higher TNM stage, (y)pT stage, and (y)pN stage were all significantly associated with poorer OS, along with age ≥ 65 years and male sex. Trial arm (SCRT vs. control) was not significantly associated with OS. In a multivariate analysis, TNM stage, age group, and sex remained independent prognostic factors (Table 2). In a multivariate analysis which included these clinicopathological factors along with TCD and a treatment interaction term, in the control arm, low whole tumour TCD was associated with a significantly higher risk of death (HR 2.03, 95% CI 1.26–3.27, *p* = 0.004). Conversely, in the SCRT arm, low whole tumour TCD was associated with a trend towards improved survival (HR 0.64, 95% CI 0.41–1.02, *p* = 0.059), with a significant interaction between TCD and trial arm (*p_interaction_* = 0.0006). Similar associations were seen with biopsy TCD and OS, although these did not reach statistical significance, presumably due to the lower number of cases included.

### 3.3. Tumour-Infiltrating Lymphocytes

#### 3.3.1. Tumour-Infiltrating Lymphocyte Density in Different Tumour Regions

**Figure 4:**
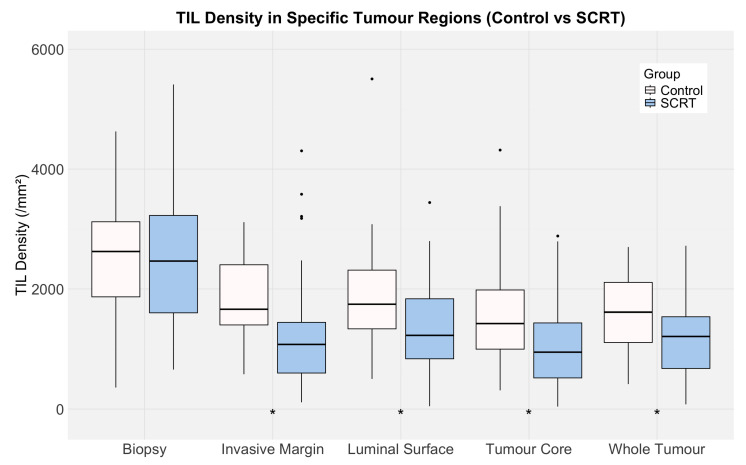
Boxplot illustrating TIL densities across the different tumour regions in control (white) and SCRT (blue) arms. The solid black line indicates the median TIL density, the box shows the interquartile range (IQR), and the whiskers represent 1.5 × IQR. Asterisks (*) denote regions where TIL densities differed significantly between arms for each region (Mann–Whitney U test, *p* < 0.05). Corresponding *p*-values: biopsy, *p* = 0.667; invasive margin, *p* < 0.001; luminal surface, *p* = 0.010; tumour core, *p* = 0.002; whole tumour, *p* = 0.001.

There was no significant difference in biopsy TIL densities between the control arm and SCRT arm (2625/mm^2^ and 2466/mm^2^, respectively, *p* = 0.667). Paired tests showed that biopsy TIL density was significantly greater than resection TIL density in all of the different resection tumour regions across both arms (*p* = 0.002 for the luminal surface in the control group, and *p* < 0.001 for all other regions in both arms). Median luminal surface TIL densities in the resection specimens (control arm = 1745/mm^2^, SCRT arm = 1229/mm^2^) were significantly lower than their respective biopsy densities (both *p* < 0.001), indicating that biopsy and resection luminal surface TIL densities are not directly comparable. In SCRT arm resections, TIL density was significantly lower across all annotated regions compared to controls (Figure 4), suggesting that radiotherapy reduces TIL density across the entire tumour.

**Figure 5:**
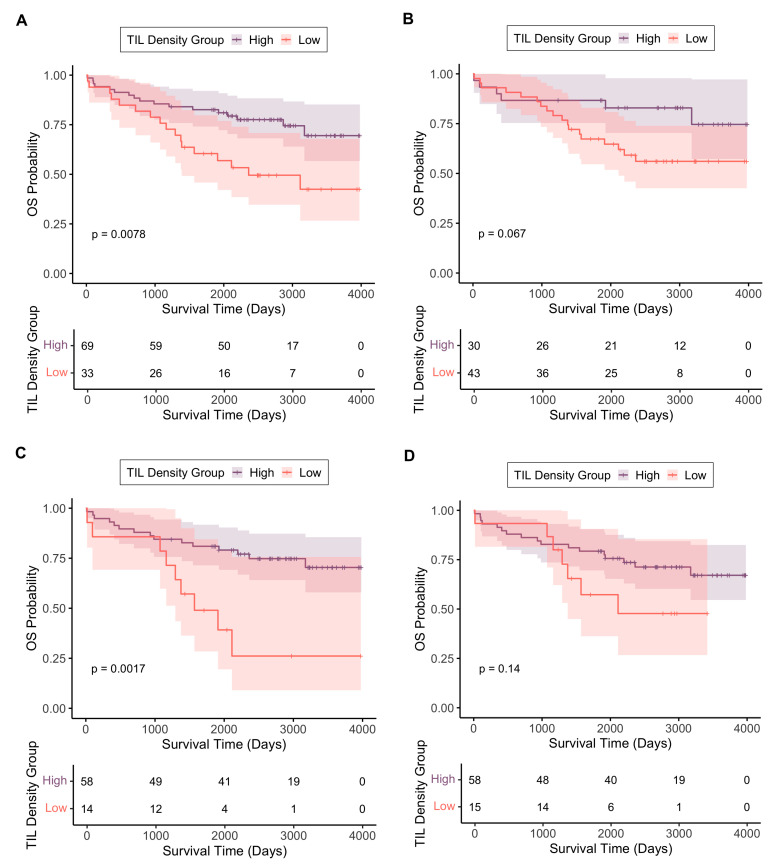

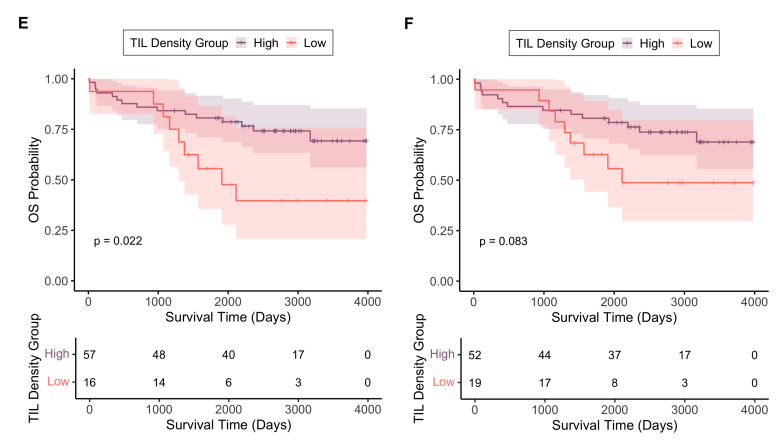
Kaplan–Meier curves showing overall survival for TIL high/low groups across the different tumour regions: (**A**) biopsy, TIL density cutoff point 1837/mm^2^ (combined control and SCRT arms); (**B**) biopsy, TIL density cutoff point 2687/mm^2^ (SCRT arm); (**C**) resection invasive margin, TIL density cutoff point 558/mm^2^ (SCRT arm); (**D**) resection luminal surface, TIL density cutoff point 731/mm^2^ (SCRT arm); (**E**) resection whole tumour area, TIL density cutoff point 598/mm^2^ (SCRT arm); (**F**) resection tumour core, TIL density cutoff point 524/mm^2^ (SCRT arm). The shaded regions around each line represent 95% confidence intervals. The *p*-values on each graph are derived from the respective log-rank test. Beneath each graph is the respective number at risk table. Time zero represents the date of surgery.

In both the control and SCRT arms, a lower biopsy TIL density was associated with poorer OS (Figure 5, both arms combined HR 2.43, 95% CI 1.24–4.76, *p* = 0.010). In the SCRT arm, lower resection TIL density at the invasive margin and in the whole tumour region was associated with poorer OS (invasive margin: HR 3.57, 95% CI 1.53–8.31, *p* = 0.0032; whole tumour: HR 2.55, 95% CI 1.11–5.87, *p* = 0.027), as seen in Figure 5. Similar but non-significant trends were seen at the luminal surface (HR 1.94, 95% CI 0.80–4.72, *p* = 0.144) and tumour core regions (HR 2.08, 95% CI 0.89–4.83, *p* = 0.090).

#### 3.3.2. TIL Density as an Independent Prognostic Biomarker

In the SCRT arm of the TIL cohort, where participants had complete clinicopathological data (n = 71), after adjusting for age, sex and TNM stage, low TIL density in the invasive margin and whole tumour regions remained associated with a higher risk of death (invasive margin HR 4.10, 95% CI 1.49–11.31, *p* = 0.006; whole tumour HR 2.82, 95% CI 1.07–7.45, *p* = 0.036). Across both arms (n = 99), a low TIL density in the biopsy was also associated with a higher risk of death in a multivariate analysis (HR 2.82, 95% CI 1.07–7.45, *p* = 0.036).

### 3.4. TIL Density and TCD

In the patients where TIL density was evaluated, in the SCRT arm, low-TCD in the resection whole tumour area remained associated with better OS (HR 0.22, 95% CI 0.084–0.568, *p* = 0.0018). In the SCRT arm, those with a low whole tumour TCD had a lower median biopsy TIL density than those with a high-TCD (2434 vs. 3219/mm^2^, *p* = 0.0766), but TIL density in the biopsies and different tumour regions showed no significant correlations with the whole tumour area resection TCD measurements. However, when evaluating both arms together, TIL density in the invasive margin region showed a significant positive correlation with whole tumour TCD (Spearman’s Rho = 0.29, *p* = 0.0033). Furthermore, when cases were dichotomised into high/low-TCD using cutoffs of 21% and 28.5% for control and SCRT arms, respectively, higher median TIL densities were observed in patients with high whole tumour TCD (TCD high = 1528/mm^2^, TCD low = 1225/mm^2^, *p* = 0.0058), tumour core (TCD high = 1413/mm^2^, TCD low = 901/mm^2^, *p* = 0.0049), invasive margin (TCD high = 1617/mm^2^, TCD low = 1054/mm^2^, *p* = 3.15 × 10^−5^), and luminal surface regions (TCD high = 1716/mm^2^, TCD low = 1212/mm^2^, *p* = 0.0289). Median TIL density in the biopsies of patients with low-TCD (2519/mm^2^) was similar to that in patients with high-TCD (2625/mm^2^, *p* = 0.4996). Biopsy TIL density in the SCRT arm was negatively correlated with the absolute change in TCD (Spearman’s Rho = −0.25, *p* = 0.043), suggesting that patients with higher TIL density prior to radiotherapy demonstrated less tumour regression.

## 4. Discussion

This study evaluated the prognostic value of TCD and TIL density in rectal cancer and explored whether they can predict response to neoadjuvant radiotherapy using pre- and post-treatment pathological specimens from patients enrolled in the MRC CR07 trial. The CR07 trial demonstrated a 61% reduction in the relative risk of local recurrence and a 24% relative improvement in disease-free survival for patients receiving SCRT when compared with selective postoperative chemoradiotherapy [5]. We show that SCRT reduces both TCD and TIL density between pre-treatment biopsy and post-treatment resection, when compared with controls. In controls, low resection TCD predicted poorer OS and CSS, consistent with previous findings in colorectal cancer [6], which suggests that tumours with a lower proportion of cancer cells may exhibit more aggressive behaviour. Conversely, low resection TCD in the SCRT group was associated with improved OS. Across the tumour bed, tumour cell destruction and replacement by fibrotic tissue occur with tumour regression after radiotherapy and greater reductions correlate with improved outcomes [23]. Thus, it is essential that the TCD measured on resection specimens is interpreted in the context of patient treatment.

In the control arm, resection TCD provides a similar measure to the tumour-to-stroma ratio. A low tumour-to-stroma ratio in colorectal cancer has been associated with worse disease-free survival and OS [24]. Our findings validate these results in a rectal cancer population. Early-phase prospective studies involving neoadjuvant radiotherapy have demonstrated that TCD in the post-treatment resection specimen can serve as a measure of treatment response, correlating well with other measures of response, including magnetic resonance imaging and the pathological tumour regression grade [25]. Retrospective exploratory studies have also employed TCD as a response marker in both short- and long-course radiotherapy [26]. Our findings reinforce its value by showing consistent associations between TCD and survival, with a decrease in resection TCD consistent with radiotherapy-induced cell death [27].

A key finding of this study is that a lower pre-treatment TCD in the diagnostic biopsy was associated with improved cancer-specific survival in patients who received radiotherapy. This is a significant result because a low-TCD, which indicates a stroma-rich tumour, is typically associated with more aggressive biology and poorer outcomes in untreated colorectal cancer. Our findings therefore suggest that patients with a stroma-rich tumour may benefit to a greater extent from radiotherapy. This highlights the potential for using pre-treatment biopsy TCD as a predictive biomarker to identify patients most likely to benefit from radiotherapy, although the observations need to be confirmed in larger prospective series. Exploratory work to determine how much tumour area is required in the biopsy to accurately determine TCD is also warranted.

In addition to the stromal and tumour cell composition reflected by TCD, the immune microenvironment is another key determinant of prognosis. Colorectal cancers with greater immune cell infiltration have consistently been associated with a better prognosis, as shown by Galon et al. [28], Pagès et al. [29], and others [7,30]. This finding has also been demonstrated in rectal cancer [31] and extends to other malignancies [32]. In colorectal cancer, both H&E-based methods and immunohistochemistry for T cell subsets have shown that greater lymphocyte infiltration confers significant survival benefit, with many of these studies focusing on the tumour core or invasive margin regions [30].

In our study of SCRT in rectal cancer, we found that radiotherapy significantly reduced TIL density across all tumour regions, likely reflecting radiation-induced cell death of both tumour and immune cells. Despite this, higher TIL density in the whole tumour and invasive margin regions in the SCRT cohort were significantly associated with better OS, suggesting that maintaining a good level of immune infiltration within the tumour microenvironment remains important for achieving survival benefit. Furthermore, when pre-treatment biopsies from both study arms were combined, lower TIL density was significantly correlated with poorer OS, indicating that pre-treatment TIL density may serve as a key prognostic marker.

Beyond prognosis, other studies have investigated the predictive value of TIL density for neoadjuvant therapy response. A recent systematic review and meta-analysis of locally advanced rectal cancer suggests that pre-treatment CD8+ TIL density may be useful for predicting sensitivity to neoadjuvant chemoradiotherapy [31]. Biomarkers for predicting response to neoadjuvant radiotherapy are comparatively understudied. A negative correlation between TIL density in pre-treatment biopsy samples and the change in tumour cell density raises the possibility of biopsy-derived measurements helping inform individual patient treatment planning; however, confirmation of these observations in independent series and exploratory work to provide a better mechanistic understanding are required. If an association is confirmed, such measurements could be valuable in the context of organ-preserving approaches such as SCRT followed by transanal endoscopic microsurgery [33].

The assessment of TIL density presents several challenges, complicating cross-study comparisons and clinical application [11]. We found that TIL density was consistently higher in pre-treatment biopsies than at the luminal surface of resection specimens; sampling and perioperative factors might influence TIL density, challenging the assumption that the biopsy and resection values are directly comparable [19]. A robust biomarker might need to extend beyond a simple total lymphocyte density. Automated detection on H&E-stained slides does not distinguish between different TIL subtypes, whose specific function is likely important. For example, radiotherapy can upregulate immune checkpoint molecules such as PD-L1, potentially driving T cell exhaustion [34]. While studies evaluating changes in ratios of specific TIL subsets could be more informative [35], this requires additional tissue and staining, prolonging turnaround times, increasing costs, and hindering clinical implementation.

Limitations to this study include the relatively small sample size, retrospective design, and low number of events for CSS analyses. Tissue blocks or slides were collected from around half of the original study sites, and matched biopsy and resection material were available from only 23 sites, which may not be representative of the entire trial. Central pathology review of cases was not built into the original trial design, and due to resource constraints, we were not able to assess the cases for additional histological features that may be associated with TILs, TCD, and radiotherapy response, including histological subtype. Detailed information about tumour height was not available due to a lack of preoperative MRI scanning during the trial. When analysing tumour resections, we found that a previously established TCD cutoff of 47% [6] was not applicable to our cohort. This is likely due to methodological differences; our analysis quantified TCD across the entire tumour area, whereas the previous study used a selected high-density area at the luminal surface. Furthermore, our study analysed a multi-centre, rectal cancer cohort, contrasting with the single-centre colorectal cancer cohort of the earlier work. Furthermore, the generalisability of our findings may be influenced by the treatment regimen. The CR07 trial employed a relatively short interval between radiotherapy and resection, and longer intervals are known to alter clinical and pathological response in rectal cancer [36]. The higher dose per fraction over a shorter timeframe characteristic of SCRT may also have a distinct impact on the tumour microenvironment compared to long-course regimens.

To build on these findings, evaluating TCD and TIL density as biomarkers in large, independent, and prospective cohorts is needed to validate their clinical utility. Future work should also focus on the specific subtype and functional status of the TILs, and our planned work in this cohort to study cell immunophenotypes will yield further insights. Longitudinal analysis of biomarkers such as TIL density and TCD could offer a more dynamic understanding of treatment response; however, study cohorts with serial biopsies are limited. Combining TCD and TIL density measurements can provide useful prognostic information [19]. The Glasgow Microenvironment Score, another composite measure, combines the tumour-to-stroma ratio and measures of peritumoural inflammation [37]; however, manual and semi-quantitative methods can be time-consuming and subject to inter-observer variability. The adoption of automated digital pathology tools could enhance the accuracy and reproducibility of TCD and TIL density measurements and facilitate their integration into existing clinical workflows [38]. In future work, we aim to automate the quantification of TCD, in addition to TIL density, as demonstrated in this study.

## 5. Conclusions

This study demonstrates that SCRT reduces both TCD and TIL density in rectal cancer, using paired diagnostic biopsies and resection specimens from a large, randomised controlled trial. By evaluating a patient cohort with lengthy clinical follow-up, pre- and post-treatment samples, and a control arm, we have robustly distinguished the prognostic and predictive value of TCD and TILs. In control patients, we show that low resection TCD is associated with poorer OS and CSS, whereas in the SCRT group, low-TCD confers a survival advantage. Crucially, we also identify low pre-treatment biopsy TCD as a potential predictive biomarker for favourable response to SCRT, offering a potential pragmatic tool for personalised treatment planning. Additionally, we show that the survival benefit from SCRT may depend not only on successful tumour cell killing but also on preserving a suitable degree of anti-tumour immune response in the form of TIL density. These insights, and future work exploring the interplay between direct cytotoxic effects and immune-mediated mechanisms in radiotherapy response, will pave the way for future biomarker-driven stratification strategies in rectal cancer.

## Figures and Tables

**Table 1:** Relationship between whole TCD and whole tumour TIL density and clinicopathological variables.

Patient Clinicopathological Characteristics		Tumour Cell Density Cohort		Median TCD (%) Across Resection Whole Tumour Area (IQR)	*p*-Value	TIL Density Cohort		Median TIL Density (/mm^2^) Across Resection Whole Tumour Area (IQR)	*p*-Value
		*n*	%			*n*	%		
All patients		569	100	27.3	n/a	102	100	1304	n/a
Sex	Male	402	70.7	26.7 (17.7–35.4)	0.10	67	65.7	1377 (835–1703)	0.75
	Female	167	28.0	28.9 (18.9–38.7)		35	34.3	1262 (900–1628)	
Age	<65 years	265	46.6	25.8 (18.1–35.7)	0.17	54	52.9	1297 (896–1607)	0.64
	≥65 years	304	53.4	28.6 (17.9–36.6)		48	47.1	1323 (819–1770)	
(y)pT	1	32	5.6	32.0 (15.4–38.3)	0.27	6	5.9	1389 (1217–1981)	0.64
	2	159	27.9	24.5 (16.0–34.8)		32	31.4	1407 (1017–1733)	
	3	335	59.2	28.0 (19.7–36.3)		56	54.9	1203 (826–1614)	
	4	38	6.7	26.6 (16.4–35.5)		8	7.8	1135 (711–1671)	
	Unknown	5	0.9	28.0 (18.3–31.9)		0	0	n/a	
(y)pN	0	310	54.5	27.3 (18.0–36.1)	0.50	59	57.8	1299 (979–1700)	0.40
	1	159	27.9	28.0 (18.3–35.6)		28	27.5	1364 (567–1626)	
	2	95	16.7	27.8 (17.8–36.9)		15	14.7	1287 (760–1632)	
	Unknown	5	0.9	28.0 (18.3–31.9)		0	0	n/a	
TNM stage	I	140	24.6	25.5 (15.4–35.5)	0.18	30	29.4	1514 (1144–2027)	0.11
	II	151	26.5	28.7 (20.8–36.2)		28	27.5	1314 (950–1621)	
	III	238	41.8	28.2 (18.6–36.2)		41	40.2	1168 (598–1615)	
	IV	4	0.7	21.7 (16.2–27.1)		0	0	n/a	
	Unknown	36	6.3	23.1 (18.1–31.4)		3	2.9	1685 (1298–1999)	
Tumour grade	Other	483	84.9	28.2 (18.8–36.6)	**0.036**	93	91.2	1349 (944–1659)	0.37
	Poorly differentiated	82	14.4	24.5 (16.2–34.2)		8	7.8	972 (708–1449)	
	Unknown	4	0.7	10.3 (4.5–15.7)		1	1.0	579	
Trial arm	SCRT	275	48.3	19.9 (12.9–26.7)	**<0.001**	73	71.6	1210 (677–1539)	**0.0013**
	Control	294	51.7	34.3 (27.7–40.5)		29	28.4	1615 (1110–2109)	

Pathological TNM staging was performed using the 5th edition [22]. Age at the time of surgery. SCRT = neoadjuvant short-course radiotherapy. n/a = not applicable. *p*-values in bold are <0.05.

**Table 2:** Univariate and multivariate analyses for overall survival in the TCD cohort.

Patient Clinicopathological Characteristics		Univariate Hazard Ratio (95% Confidence Interval)	*p*-Value	Multivariate Hazard Ratio (95% Confidence Interval)	*p*-Value
Sex	Female	1.00		1.00	
	Male	1.50 (1.09–2.07)	**0.01**	1.53 (1.11–2.11)	**0.009**
Age	<65 years	1.00		1.00	
	≥65 years	2.24 (1.68–2.98)	**<0.001**	2.33 (1.74–3.11)	**<0.001**
(y)pT	1	1.00		n/a	n/a
	2	1.31 (0.59–2.92)	0.514		
	3	2.44 (1.14–5.22)	**0.021**		
	4	5.63 (2.43–13.05)	**<0.001**		
(y)pN	0	1.00		n/a	n/a
	1	1.32 (0.97–1.81)	0.082		
	2	2.10 (1.50–2.94)	**<0.001**		
TNM stage	I	1.00		1.00	
	II	1.33 (0.89–1.99)	0.165	1.31 (0.87–1.97)	0.196
	III	1.77 (1.23–2.54)	**0.002**	1.84 (1.28–2.65)	**<0.001**
	IV	5.92 (2.11–16.58)	**<0.001**	7.13 (2.48–20.47)	**<0.001**
Tumour grade	Other	1.00		1.00	
	Poorly differentiated	1.28 (0.90–1.83)	0.176	1.14 (0.79–1.65)	0.489
Trial arm	Control	1.00		1.00	
	SCRT	0.93 (0.71–1.22)	0.595	0.96 (0.73–1.26)	0.747

Pathological TNM staging was performed using the 5th edition [22]. Age at the time of surgery. SCRT = neoadjuvant short-course radiotherapy. n/a = not applicable, as integrated TNM stage is included instead. N = 530 (39 cases excluded due to missing stage/grade data). *p*-values in bold are <0.05.

## Data Availability

The data that support the findings of this study are not openly available, but are available from the corresponding author upon reasonable request. Data are located in controlled access data storage at the University of Leeds.
